# Supplementary material for: Individual differences in functional brain connectivity predict temporal discounting preference in the transition to adolescence
Source: Dev Cogn Neurosci. 2018 Jul 30;34:101–13. doi: 10.1016/j.dcn.2018.07.003 (PMC6969312; doi:10.1016/j.dcn.2018.07.003)
Supplement: Supplementary file 1 [file mmc1.docx]

**Supplementary Information**

Supplementary Methods and Results

**Supplemental Table 3** presents the model characteristics for the nine connections of interest identified in this study with and without the inclusion of key covariates, for each sample. The first set of models presented are for the longitudinal sample. Beneath the first set of models are the models for the cross-sectional (replication) sample. The first set of model parameters presented for each sample are the results presented in the main manuscript’s **Table 4**, the best fitting models without the inclusion of covariates (no covariate model). To account for the potential impact of IQ on these models, we then included the participant’s IQ as a main effect in the identified best fitting model. The model parameters for this “IQ covariate model” are presented in the second set of model parameters. For the longitudinal sample, we conducted a likelihood ratio test to compare the “no covariate model” with the IQ covariate model, and the resulting LR test statistics are presented here, as well as the difference in AIC between the two models. IQ did not improve any of the longitudinal models, and the effect size (unstandardized estimate) for IQ was 0. Further, the estimates for connectivity were essentially unchanged in this IQ covariate model. For the cross-sectional sample, we conducted an F test to assess the impact of including IQ as a covariate in the model. For each connection of interest, including IQ improved the model, however its impact on the overall model (effect size) was small. Importantly, the inclusion of IQ in these models did not impact the effect size of the connectivity measure.

We also assessed the impact of puberty (assessed via Tanner staging) and race (white vs. non-white) on our models. Because we were missing some of these variables for some of our participants (see table below) these covariates were examined separately from each other, as well as from IQ.

|  | Original sample  N (scans) | Original Sample w/ IQ covariate  N (scans) | Original Sample w/ Race covariate  N (scans) | Original Sample w/ Puberty covariate  N (scans) |
| --- | --- | --- | --- | --- |
| Sample 1 | 64 (137) | 64 (137) | 49 (104) | 33 (69) |
| Sample 2 | 84 | 84 | 40 | 26 |

Our ability to examine the impact of puberty or race on our results was limited given the reduction in sample size. Further, the correlation between our pubertal measure (Tanner staging) and age was r = .52 p < 0.0001 (see scatter plot of puberty and age for the two samples combined below). The majority of two samples identified as white (sample 1: 64% white, 12.5% non-white, 23.5% no data; sample 2: 35.7% white, 12% non-white, 52.3% no data). Nevertheless, the inclusion of any of these covariates (puberty or race) in the best fitting models identified in the original analysis are presented in Supplementary Table 3.


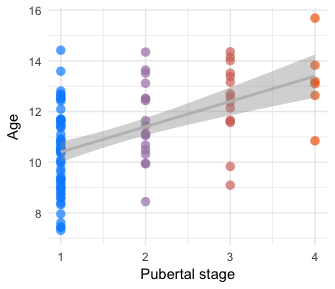


We also examined if variance in a participant’s temporal discounting preference at the second time point of assessment (in the longitudinal sample) could be explained by changes in functional connectivity between the two time points (controlling for baseline temporal discounting preference) using linear regression. We also assessed if the prediction of temporal discounting preference was improved if we included an interaction effect between changes in functional connectivity and the age of the participant at the second timepoint. Changes in connectivity between three out of the nine relevant connections were able to explain variance in future temporal discounting after controlling for baseline temporal discounting preference (see **Supplementary Table 4**). Increased strength between the left dlPFC and right dACC across time was associated with increased preference for waiting for later larger rewards, whereas the opposite developmental finding was observed for connectivity between the right PCC and right pallidum. This is in line with our finding that increased strength between cognitive control regions, and decreased strength between valuation system regions, is related to increased preference for later larger rewards in the transition into adolescence. Further, developmental changes in connectivity strength between the left amygdala and right OFC interacted with the age of the participant at the second timepoint to explain variance in future temporal discounting after controlling for baseline temporal discounting preference. While increased strength between the left amygdala and right OFC across time was generally related to increased preference for later larger rewards, this negatively interacted with age at the second time point. This suggests that the strengthening of these two valuation regions is related to increased preference for later larger rewards for younger participants. We have included figures below illustrating the predictive value of the three models described above. The y-axis displays the unstandardized predicted value of the regression model with AUC, and changes in connectivity in the first two models, as well as Age at T2 interacting with changes in connectivity for the third graph depicting the left amygdala and right OFC connection.


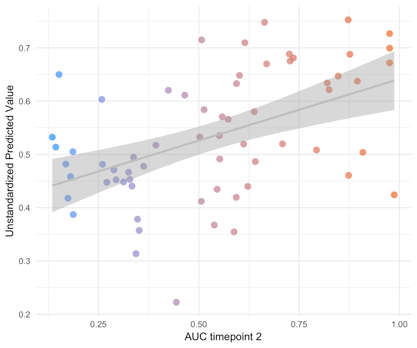

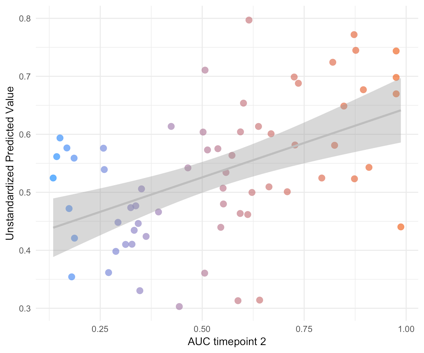

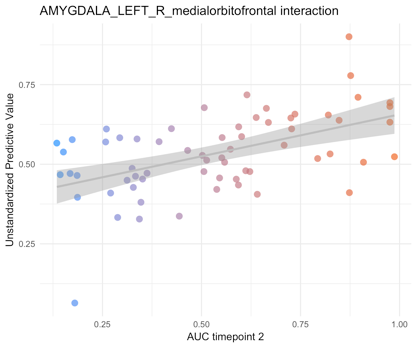


We also examined if there was a relationship between age and connectivity changes in a combined sample of both the longitudinal and cross-sectional samples. Because these participants were acquired with the same protocol and on the same MRI scanner, we pooled them to investigate the relationship between connectivity our nine connections of interest and age. We used mixed-effects modeling and compared linear, quadratic, and cubic age models with a null model including only the random intercept for each participant. Of the nine connections, only the connection between the left dlPFC and right PCC showed an age-relationship (linear). This graph is included below.


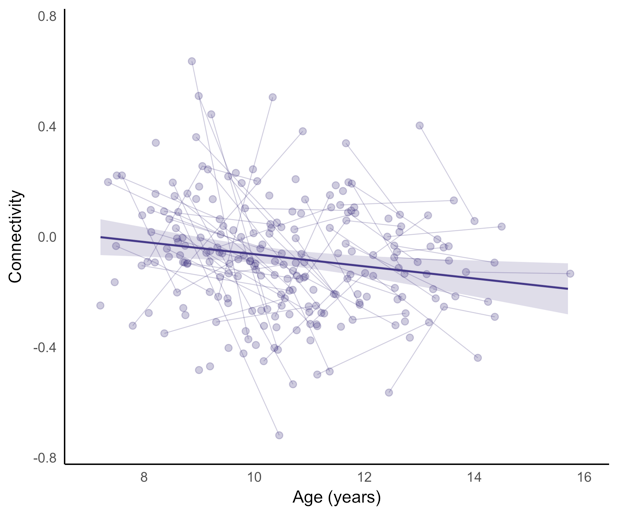


Supplementary Table 1. Regions of interest in the present study.

| **Parcel network** | **Hemisphere** | **Parcel FreeSurfer label** | **Parcel colloquial name** | **Parcel colloquial name (specific)** |
| --- | --- | --- | --- | --- |
| control | left | L_caudalanteriorcingulate | dACC | dorsal ACC |
| control | right | R_caudalanteriorcingulate | dACC | dorsal ACC |
| control | left | L_caudalmiddlefrontal | dlPFC | dlPFC |
| control | right | R_caudalmiddlefrontal | dlPFC | dlPFC |
| valuation | left | L_isthmuscingulate | PCC | isthmus cingulate |
| valuation | right | R_isthmuscingulate | PCC | isthmus cingulate |
| valuation | left | L_lateralorbitofrontal | lOFC | lateral OFC |
| valuation | right | R_lateralorbitofrontal | lOFC | lateral OFC |
| valuation | left | L_medialorbitofrontal | mOFC | medial OFC |
| valuation | right | R_medialorbitofrontal | mOFC | medial OFC |
| SMA | left | L_paracentral | SMA | SMA |
| SMA | right | R_paracentral | SMA | SMA |
| control | left | L_parsopercularis | IFG | pars opercularis |
| control | right | R_parsopercularis | IFG | pars opercularis |
| control | left | L_parsorbitalis | IFG | vlPFC |
| control | right | R_parsorbitalis | IFG | vlPFC |
| control | left | L_parstriangularis | IFG | pars triangularis |
| control | right | R_parstriangularis | IFG | pars triangularis |
| valuation | left | L_posteriorcingulate | PCC | PCC |
| valuation | right | R_posteriorcingulate | PCC | PCC |
| valuation | left | L_precuneus | PCC | precuneus |
| valuation | right | R_precuneus | PCC | precuneus |
| control | left | L_rostralanteriorcingulate | rACC | rostral ACC |
| control | right | R_rostralanteriorcingulate | rACC | rostral ACC |
| control | left | L_rostralmiddlefrontal | dlPFC | rostral middle frontal cortex |
| control | right | R_rostralmiddlefrontal | dlPFC | rostral middle frontal cortex |
| control | left | L_superiorfrontal | dlPFC | superior frontal cortex |
| control | right | R_superiorfrontal | dlPFC | superior frontal cortex |
| control | left | L_frontalpole | dlPFC | frontal pole |
| control | right | R_frontalpole | dlPFC | frontal pole |
| control | left | CAUDATE_LEFT | dorsal striatum | caudate |
| control | right | CAUDATE_RIGHT | dorsal striatum | caudate |
| control | left | PUTAMEN_LEFT | dorsal striatum | putamen |
| control | right | PUTAMEN_RIGHT | dorsal striatum | putamen |
| valuation | left | PALLIDUM_LEFT | ventral striatum | pallidum |
| valuation | right | PALLIDUM_RIGHT | ventral striatum | pallidum |
| hippocampus | left | HIPPOCAMPUS_LEFT | hippocampus | hippocampus |
| hippocampus | right | HIPPOCAMPUS_RIGHT | hippocampus | hippocampus |
| valuation | left | AMYGDALA_LEFT | amygdala | amygdala |
| valuation | right | AMYGDALA_RIGHT | amygdala | amygdala |
| valuation | left | ACCUMBENS_LEFT | ventral striatum | NAcc |
| valuation | right | ACCUMBENS_RIGHT | ventral striatum | NAcc |

Supplementary Table 2. Connections that significantly improved the age-only model for predicting AUC in the longitudinal sample.

| **Connection of Interest** | **Network - Network** | **Best Fit Model** | **Likelihood Ratio test** | **AIC difference** | **Intercept Estimate (SE)** | **Linear age Estimate (SE)** | **Quadratic age Estimate (SE)** | **Connectivity Estimate (SE)** | **Linear age X Connectivity Estimate (SE)** | **Quadratic age X Connectivity Estimate (SE)** |
| --- | --- | --- | --- | --- | --- | --- | --- | --- | --- | --- |
| Left vlPFC – Right Rostral Middle Frontal Cortex | Control – Control | main effect | *X^2^*(1) = 14.37, p = 0.0002 | 12.37 | 0.54 (0.03) | 0.05 (0.01) | -0.01 (0.01) | 0.4 (0.1) | - | - |
| Left Hippocampus – Left SMA | Hippocampus – SMA | linear interaction | *X^2^*(2) = 15.21, p = 0.0005 | 11.21 | 0.56 (0.03) | 0.05 (0.01) | -0.01 (0.01) | 0.12 (0.13) | -0.25 (0.08) | - |
| Left Hippocampus – Right SMA | Hippocampus – SMA | linear interaction | *X^2^*(2) = 14.54, p = 0.0007 | 10.54 | 0.56 (0.03) | 0.04 (0.01) | -0.01 (0.01) | 0.16 (0.13) | -0.23 (0.08) | - |
| Left NAcc – Right SMA | Valuation – SMA | linear interaction | *X^2^*(2) = 14.03, p = 0.0009 | 10.03 | 0.52 (0.03) | 0.01 (0.01) | -0.01 (0.01) | -0.34 (0.14) | -0.32 (0.09) | - |
| Left mOFC – Right Pars Triangularis | Valuation – Control | main effect | *X^2^*(1) = 11.03, p = 0.0009 | 9.03 | 0.58 (0.03) | 0.05 (0.01) | -0.01 (0.01) | 0.36 (0.1) | - | - |
| Left PCC – Right Pars Triangularis | Valuation – Control | quadratic interaction | *X^2^*(2) = 12.99, p = 0.0015 | 8.99 | 0.54 (0.03) | 0.05 (0.01) | -0.01 (0.01) | -0.41 (0.15) | - | 0.1 (0.03) |
| Left lOFC – Right Rostral Middle Frontal Cortex | Valuation – Control | main effect | *X^2^*(1) = 10.96, p = 0.0009 | 8.96 | 0.51 (0.03) | 0.04 (0.01) | -0.01 (0.01) | 0.38 (0.11) | - | - |
| Right Hippocampus – Left SMA | Hippocampus – SMA | quadratic interaction | *X^2^*(2) = 12.58, p = 0.0019 | 8.58 | 0.56 (0.03) | 0.04 (0.01) | -0.01 (0.01) | -0.01 (0.15) | - | 0.12 (0.04) |
| Left Rostral Middle Frontal Cortex – Right Rostral Middle Frontal Cortex | Control – Control | main effect | *X^2^*(1) = 10.51, p = 0.0012 | 8.51 | 0.32 (0.08) | 0.05 (0.01) | -0.01 (0.01) | 0.46 (0.14) | - | - |
| Left Accumbens – Left Isthmus Cingulate | Valuation – Valuation | linear interaction | *X^2^*(2) = 12.24, p = 0.0022 | 8.24 | 0.55 (0.03) | 0.04 (0.01) | -0.01 (0.01) | 0.03 (0.13) | 0.24 (0.08) | - |
| Right Putamen – Right PCC | Control – Valuation | main effect | *X^2^*(1) = 10.13, p = 0.0015 | 8.13 | 0.57 (0.03) | 0.04 (0.01) | -0.01 (0.01) | -0.38 (0.12) | - | - |
| Right Amygdala – Right rACC | Valuation – Control | main effect | *X^2^*(1) = 9.96, p = 0.0016 | 7.96 | 0.57 (0.03) | 0.04 (0.01) | -0.01 (0.01) | 0.37 (0.11) | - | - |
| Left lOFC – Right Superior Frontal Cortex | Valuation – Control | main effect | *X^2^*(1) = 9.91, p = 0.0016 | 7.91 | 0.52 (0.03) | 0.04 (0.01) | -0.01 (0.01) | 0.33 (0.1) | - | - |
| Left Caudate – Left Pars Triangularis | Control – Control | main effect | *X^2^*(1) = 9.88, p = 0.0017 | 7.88 | 0.64 (0.04) | 0.04 (0.01) | -0.01 (0.01) | -0.37 (0.12) | - | - |
| Right Hippocampus – Right SMA | Hippocampus – SMA | quadratic interaction | *X^2^*(2) = 11.79, p = 0.0027 | 7.79 | 0.56 (0.03) | 0.04 (0.01) | -0.01 (0) | -0.08 (0.18) | - | 0.14 (0.05) |
| Left Pars Triangularis – Right rACC | Control – Control | main effect | *X^2^*(1) = 9.64, p = 0.0019 | 7.64 | 0.55 (0.03) | 0.04 (0.01) | -0.01 (0.01) | 0.33 (0.11) | - | - |
| Left rACC – Right vlPFC | Control – Control | main effect | *X^2^*(1) = 9.61, p = 0.0019 | 7.61 | 0.53 (0.03) | 0.04 (0.01) | -0.01 (0.01) | 0.34 (0.1) | - | - |
| Left vlPFC – Right Superior Frontal Cortex | Control – Control | main effect | *X^2^*(1) = 9.49, p = 0.0021 | 7.49 | 0.52 (0.03) | 0.04 (0.01) | -0.01 (0.01) | 0.34 (0.11) | - | - |
| Left dlPFC – Right PCC | Control – Valuation | quadratic interaction | *X^2^*(2) = 11.44, p = 0.0033 | 7.44 | 0.54 (0.03) | 0.06 (0.01) | -0.01 (0.01) | -0.08 (0.11) | - | 0.1 (0.03) |
| Left Superior Frontal Cortex – Right Rostral Middle Frontal Cortex | Control – Control | main effect | *X^2^*(1) = 9.2, p = 0.0024 | 7.2 | 0.55 (0.03) | 0.05 (0.01) | -0.01 (0.01) | 0.27 (0.09) | - | - |
| Left lOFC – Right vlPFC | Valuation – Control | main effect | *X^2^*(1) = 9.09, p = 0.0026 | 7.09 | 0.43 (0.05) | 0.04 (0.01) | -0.01 (0.01) | 0.36 (0.12) | - | - |
| Left Superior Frontal Cortex – Right Superior Frontal Cortex | Control – Control | main effect | *X^2^*(1) = 8.95, p = 0.0028 | 6.95 | 0.36 (0.07) | 0.05 (0.01) | -0.01 (0.01) | 0.37 (0.12) | - | - |
| Left Pallidum – Right Pars Triangularis | Valuation – Control | quadratic interaction | *X^2^*(2) = 10.92, p = 0.0042 | 6.92 | 0.53 (0.03) | 0.05 (0.01) | -0.01 (0.01) | 0.44 (0.17) | - | -0.1 (0.03) |
| Right Pars Triangularis – Right PCC | Control – Valuation | quadratic interaction | *X^2^*(2) = 10.87, p = 0.0044 | 6.87 | 0.57 (0.03) | 0.04 (0.01) | -0.02 (0.01) | -0.45 (0.14) | - | 0.09 (0.03) |
| Right Isthmus Cingulate – Right Precuneus | Valuation – Valuation | main effect | *X^2^*(1) = 8.76, p = 0.0031 | 6.76 | 0.78 (0.08) | 0.04 (0.01) | -0.01 (0.01) | -0.37 (0.12) | - | - |
| Left PCC – Right Pallidum | Valuation – Valuation | main effect | *X^2^*(1) = 8.74, p = 0.0031 | 6.74 | 0.54 (0.03) | 0.05 (0.01) | -0.01 (0.01) | -0.38 (0.12) | - | - |
| Left dlPFC – Right dlPFC | Control – Control | main effect | *X^2^*(1) = 8.68, p = 0.0032 | 6.68 | 0.4 (0.06) | 0.05 (0.01) | -0.01 (0.01) | 0.36 (0.12) | - | - |
| Left dACC – Left rACC | Control – Control | main effect | *X^2^*(1) = 8.46, p = 0.0036 | 6.46 | 0.48 (0.04) | 0.04 (0.01) | -0.01 (0.01) | 0.36 (0.12) | - | - |
| Left Amygdala – Right rACC | Valuation – Control | main effect | *X^2^*(1) = 8.29, p = 0.004 | 6.29 | 0.57 (0.03) | 0.04 (0.01) | -0.01 (0.01) | 0.34 (0.12) | - | - |
| Right Pallidum – Right PCC | Valuation – Valuation | main effect | *X^2^*(1) = 8.27, p = 0.004 | 6.27 | 0.56 (0.03) | 0.05 (0.01) | -0.01 (0.01) | -0.34 (0.11) | - | - |
| Left Amygdala – Left SMA | Valuation – SMA | linear interaction | *X^2^*(2) = 10.13, p = 0.0063 | 6.13 | 0.55 (0.03) | 0.05 (0.01) | -0.01 (0.01) | 0.12 (0.13) | -0.2 (0.08) | - |
| Right NAcc – Right Pars Opercularis | Valuation – Control | linear interaction | *X^2^*(2) = 10.11, p = 0.0064 | 6.11 | 0.56 (0.03) | 0.03 (0.01) | -0.01 (0.01) | 0.13 (0.12) | -0.19 (0.07) | - |
| Left vlPFC – Right Pars Opercularis | Control – Control | main effect | *X^2^*(1) = 8.11, p = 0.0044 | 6.11 | 0.6 (0.03) | 0.04 (0.01) | -0.01 (0.01) | 0.36 (0.12) | - | - |
| Left Pars Triangularis – Left Putamen | Control – Control | main effect | *X^2^*(1) = 8.07, p = 0.0045 | 6.07 | 0.61 (0.04) | 0.04 (0.01) | -0.01 (0.01) | -0.37 (0.13) | - | - |
| Left PCC – Right Putamen | Valuation – Control | main effect | *X^2^*(1) = 8.01, p = 0.0046 | 6.01 | 0.56 (0.03) | 0.04 (0.01) | -0.01 (0.01) | -0.38 (0.13) | - | - |
| Left Superior Frontal Cortex – Right Pars Triangularis | Control – Control | main effect | *X^2^*(1) = 7.97, p = 0.0048 | 5.97 | 0.55 (0.03) | 0.04 (0.01) | -0.01 (0.01) | 0.28 (0.1) | - | - |
| Left Amygdala – Right mOFC | Valuation – Valuation | quadratic interaction | *X^2^*(2) = 9.97, p = 0.0069 | 5.97 | 0.59 (0.04) | 0.04 (0.01) | -0.02 (0.01) | -0.23 (0.15) | - | 0.09 (0.03) |
| Left Superior Frontal Cortex – Right PCC | Control – Valuation | quadratic interaction | *X^2^*(2) = 9.9, p = 0.0071 | 5.9 | 0.55 (0.03) | 0.06 (0.01) | -0.01 (0.01) | -0.2 (0.12) | - | 0.1 (0.03) |
| Right Amygdala – Right Pars Triangularis | Valuation – Control | main effect | *X^2^*(1) = 7.87, p = 0.005 | 5.87 | 0.57 (0.03) | 0.04 (0.01) | -0.01 (0.01) | 0.31 (0.11) | - | - |
| Left dACC – Right Rostral Middle Frontal Cortex | Control – Control | main effect | *X^2^*(1) = 7.85, p = 0.0051 | 5.85 | 0.51 (0.03) | 0.04 (0.01) | -0.01 (0.01) | 0.29 (0.1) | - | - |
| Left Pars Triangularis – Right Superior Frontal Cortex | Control – Control | main effect | *X^2^*(1) = 7.69, p = 0.0056 | 5.69 | 0.55 (0.03) | 0.04 (0.01) | -0.01 (0.01) | 0.31 (0.11) | - | - |
| Left Pallidum – Right Isthmus Cingulate | Valuation – Valuation | main effect | *X^2^*(1) = 7.56, p = 0.006 | 5.56 | 0.58 (0.03) | 0.05 (0.01) | -0.01 (0.01) | 0.35 (0.12) | - | - |
| Right NAcc – Right paracentral | Valuation – SMA | linear interaction | *X^2^*(2) = 9.51, p = 0.0086 | 5.51 | 0.54 (0.03) | 0.03 (0.01) | -0.01 (0.01) | -0.18 (0.13) | -0.24 (0.08) | - |
| Left Pars Triangularis – Left Rostral Middle Frontal Cortex | Control – Control | linear interaction | *X^2^*(2) = 9.51, p = 0.0086 | 5.51 | 0.59 (0.04) | 0.1 (0.02) | -0.01 (0.01) | -0.1 (0.1) | -0.2 (0.06) | - |
| Left dlPFC – Right dACC | Control – Control | main effect | *X^2^*(1) = 7.13, p = 0.0076 | 5.13 | 0.59 (0.03) | 0.05 (0.01) | -0.01 (0.01) | 0.26 (0.1) | - | - |
| Left mOFC – Right vlPFC | Valuation – Control | main effect | *X^2^*(1) = 7.13, p = 0.0076 | 5.13 | 0.51 (0.04) | 0.04 (0.01) | -0.01 (0.01) | 0.27 (0.1) | - | - |
| Right Frontal Pole – Right rACC | Control – Control | main effect | *X^2^*(1) = 7.11, p = 0.0077 | 5.11 | 0.52 (0.03) | 0.04 (0.01) | -0.01 (0.01) | 0.27 (0.1) | - | - |
| Right Amygdala – Left rACC | Valuation – Control | main effect | *X^2^*(1) = 7.04, p = 0.008 | 5.04 | 0.56 (0.03) | 0.04 (0.01) | -0.01 (0.01) | 0.34 (0.13) | - | - |
| Right Precuneus – Right Superior Frontal Cortex | Valuation – Control | main effect | *X^2^*(1) = 6.92, p = 0.0085 | 4.92 | 0.58 (0.03) | 0.04 (0.01) | -0.01 (0.01) | -0.28 (0.11) | - | - |
| Left vlPFC – Right dlPFC | Control – Control | main effect | *X^2^*(1) = 6.89, p = 0.0087 | 4.89 | 0.55 (0.03) | 0.04 (0.01) | -0.01 (0.01) | 0.29 (0.11) | - | - |
| Left lOFC – Left rACC | Valuation – Control | main effect | *X^2^*(1) = 6.81, p = 0.0091 | 4.81 | 0.44 (0.05) | 0.04 (0.01) | -0.01 (0.01) | 0.35 (0.13) | - | - |
| Left dlPFC – Right Rostral Middle Frontal Cortex | Control – Control | main effect | *X^2^*(1) = 6.8, p = 0.0091 | 4.8 | 0.51 (0.04) | 0.05 (0.01) | -0.01 (0.01) | 0.29 (0.11) | - | - |
| Right Caudate – Left Pars Triangularis | Control – Control | main effect | *X^2^*(1) = 6.64, p = 0.01 | 4.64 | 0.6 (0.04) | 0.05 (0.01) | -0.01 (0.01) | -0.33 (0.13) | - | - |
| Left PCC – Right dACC | Valuation – Control | coi only | *X^2^*(-1) = 6.43, p = 0.0112 | 4.43 | 0.41 (0.05) | - | - | 0.37 (0.14) | - | - |
| Right Precuneus – Right rACC | Valuation – Control | coi only | *X^2^*(-1) = 6.32, p = 0.012 | 4.32 | 0.57 (0.03) | - | - | -0.32 (0.12) | - | - |
| Left Pars Opercularis – Right Rostral Middle Frontal Cortex | Control – Control | coi only | *X^2^*(-1) = 6.3, p = 0.0121 | 4.3 | 0.49 (0.03) | - | - | 0.33 (0.12) | - | - |
| Left Hippocampus – Right Pallidum | Hippocampus – Valuation | coi only | *X^2^*(-1) = 6.26, p = 0.0123 | 4.26 | 0.55 (0.03) | - | - | -0.31 (0.12) | - | - |
| Left Rostral Middle Frontal Cortex – Right Frontal Pole | Control – Control | coi only | *X^2^*(-1) = 5.97, p = 0.0146 | 3.97 | 0.52 (0.03) | - | - | -0.25 (0.09) | - | - |

Supplementary Table 3. Model characteristics for the nine connections of interest that replicated across the both samples with and without the inclusion of covariates. (see attached Excel file)

Supplementary Table 4. Linear regression predicting temporal discounting preference (AUC) at T2 using AUC at T1, and change in connectivity between COI between both timepoints. One COI includes an interaction between change in connectivity between both time points and age at T2.

| **Left dlPFC - Right dACC** | | | | |
| --- | --- | --- | --- | --- |
|  | Estimate | SE | t value | Pr(>\|t\|) |
| (Intercept) | 0.37 | 0.05 | 6.9 | 0.0000 |
| AUC1 | 0.37 | 0.10 | 3.9 | 0.0002 |
| changecor | 0.21 | 0.10 | 2.0 | 0.0477 |
|  |  |  |  |  |
| F-statistic: | 9.175 (2,61) p = 0.05 | | |  |
| Adjusted R-squared: | 0.206 |  |  |  |
|  |  |  |  |  |
| **Left amygdala - Right mOFC** | | | | |
|  | Estimate | SE | t value | Pr(>\|t\|) |
| (Intercept) | 0.38 | 0.20 | 1.9 | 0.0573 |
| AUC1 | 0.35 | 0.10 | 3.6 | 0.0006 |
| changecor | 1.65 | 0.69 | 2.4 | 0.0207 |
| Discounting_Age2 | 0.00 | 0.02 | -0.1 | 0.9463 |
| changecor * Discounting_Age2 | -0.13 | 0.06 | -2.2 | 0.0320 |
|  |  |  |  |  |
| F-statistic: | 5.272 (4,59) p = 0.03 | | |  |
| Adjusted R-squared: | 0.2134 |  |  |  |
|  |  |  |  |  |
| **Right PCC - Right pallidum** | | | | |
|  | Estimate | SE | t value | Pr(>\|t\|) |
| (Intercept) | 0.36 | 0.05 | 6.8 | 0.0000 |
| AUC1 | 0.40 | 0.10 | 4.1 | 0.0001 |
| changecor | -0.27 | 0.12 | -2.2 | 0.0355 |
|  |  |  |  |  |
| F-statistic: | 9.505 (2,61) p= 0.04 | | |  |
| Adjusted R-squared: | 0.2126 |  |  |  |
